# Ants of Mexico: Distribution and species richness in environments with varying levels of human impact

**DOI:** 10.3897/BDJ.11.e109794

**Published:** 2023-10-02

**Authors:** Itzel Rubí Rodríguez de León, Crystian S. Venegas Barrera, Griselda Gaona García, Ausencio Azuara Domínguez, Madai Rosas Mejía

**Affiliations:** 1 Universidad Autónoma de Tamaulipas, Instituto de Ecología Aplicada, Cd. Victoria, Mexico Universidad Autónoma de Tamaulipas, Instituto de Ecología Aplicada Cd. Victoria Mexico; 2 División de Estudios de Posgrado e Investigación, Tecnológico Nacional de México, Cd. Victoria, Mexico División de Estudios de Posgrado e Investigación, Tecnológico Nacional de México Cd. Victoria Mexico; 3 Universidad Autónoma de Tamaulipas. Facultad de Medicina Veterinaria y Zootecnia, “Dr. Norberto Treviño Zapata”, Cd. Victoria, Mexico Universidad Autónoma de Tamaulipas. Facultad de Medicina Veterinaria y Zootecnia, “Dr. Norberto Treviño Zapata” Cd. Victoria Mexico

**Keywords:** Formicidae, economic importance, health, ecology

## Abstract

**Background:**

Anthropogenic disturbance, primarily driven by land-use changes, has caused alterations in ecosystems and biodiversity, including the ant community. Therefore, the aim of this study was to analyse the current landscape of ant species richness and distribution in environments with varying degrees of disturbance in Mexico. Additionally, we sought to identify ant species of ecological, economic and health significance within the country.

**New information:**

The present study shows that Mexico has a total of 33,286 records of 1,104 ant species belonging to 10 subfamilies. These species were recorded in a wide variety of environments with different levels of human impact. It was observed that both highly-disturbed environments and undisturbed environments had the highest number of ant records. In undisturbed environments, greater species richness was recorded, with a total of 704 species. Furthermore, the most representative ant species for the country were identified in ecological, economic and human health contexts. Within these species, a group composed of four exotic species (*Tapinomamelanocephalum*, *Paratrechinalongicornis*, *Wasmanniaauropunctata* and *Linepithemahumile*) deserves special attention, as they have achieved extensive dispersion throughout the country and have been associated with negative impacts in ecological, economic and human health realms.

## Introduction

Ants are one of the most successful groups of insects in terrestrial environments, with nearly 13,000 species described worldwide and 887 species reported in Mexico (Dáttilo et al. 2020). They hold significant importance within ecosystems, participating in various ecological processes, such as seed dispersal (Hölldobler and Wilson 1990), organic matter decomposition, and nutrient recycling, which are essential for soil fertility and plant growth (Andersen and Sparling 1997). Additionally, they play a role in the pollination process of plants such as cacti and orchids. Furthermore, ants are involved in a wide range of trophic interactions, as they have a broad feeding spectrum and associate with numerous species (Redolfi et al. 2004, Parker and Kronauer 2021). From an economic standpoint, certain species are considered pests, with a negative impact on crops, while others are used to control pests and improve soil quality (Rojas-Fernández 2001, Rojas-Fernández 2011, Murguía-González et al. 2018), reducing the need for pesticide use. On the other hand, some ant species have medical significance due to their potential as carriers of pathogens (Olivera and Porras-Villamil 2021). Nevertheless, certain ant species are of importance in the pharmaceutical industry because they contain various compounds, such as antimicrobial peptides, biogenic amines, alkaloids and flavonoids, which are used for the treatment of various diseases, including asthma, cancer and microbial infections (Agarwal et al. 2022).

In Mexico, ants are particularly diverse due to topographic complexity, climatic diversity and the convergence of two biogeographic zones (Nearctic and Neotropical) (Halffter and Morrone 2017, Dáttilo et al. 2020). However, in recent years, approximately 50% of natural ecosystems have been lost due to the impact of human activities (González-Abraham et al. 2015). The areas with the highest potential for productivity and greatest accessibility are the first to be utilized for human benefit. The expansion of agricultural frontiers, livestock farming (Aguilar et al. 2000), deforestation (Palacio-Prieto et al. 2000) and human settlements are the main activities leading to the loss of the original environment, increased isolation, and reduction of remaining areas (Lozano et al. 2009). These changes have repercussions for the physical environment, causing indirect effects on the composition, abundance, and dispersal of many species (Bustamante and Grez 1995), resulting in alterations in biological interactions, increased occurrence of pests and a decline in crop pollination (Buczkowski and Richmond 2012). Furthermore, human-induced disturbance favors the establishment of exotic and invasive ant species that can displacenative species (Sobrinho and Schoereder 2007, Rosas-Mejía et al. 2021).

The diversity and distribution of ants in Mexico have been extensively studied and documented (Vásquez-Bolaños 2015, Dáttilo et al. 2020, Rocha-Ortega et al. 2023). However, there is a need for a comprehensive analysis of ant distributions in environments with varying degrees of disturbance throughout Mexico. This research aims to review the current status of these dispersal patterns of ant species in environments with different degrees of disturbance in Mexico, as well as to highlight the species of ecological, economic and health importance to humans present in the country.

## General description

### Purpose

This study provides information on the current status of the diversity and distribution of ant species in environments with varying degrees of disturbance in Mexico, collected between 1700 and 2022. These data were obtained from the Global Biodiversity Information Facility (GBIF), as well as entomological collections in Mexico. The review highlights the significance of the most representative species in the country in terms of ecological, economic and human health impacts.

## Sampling methods

### Sampling description

The assessment of the current status of ant species diversity and dispersion in environments with varying degrees of anthropogenic influence in Mexico was conducted, based on documented records from 1700 to 2022. Ant species records were obtained from the GBIF database and the National Collection of Insects at the Institute of Biology, National Autonomous University of Mexico, as well as collections from the Institute of Applied Ecology at the Autonomous University of Tamaulipas and the National Technological Institute of Mexico, Victoria Campus, Tamaulipas. The localities and geographic coordinates were verified using the Google Earth programme. Duplicate records, non-georeferenced records or records for which the latitude and longitude coordinates had fewer than two decimal places (0.0) were eliminated during the classification process. Additionally, a review of current taxon status was conducted to verify the validity of each name, grouping taxa treated as synonyms of another species with the taxon with the validated current name (Bolton 2023, AntWeb 2022). With the obtained information, two maps were created using ArcGIS 10.8 software (ESRI (Environmental Systems Research Institute) 2020):

1. to represent the dispersion of ant species in Mexico;
2. to determine the degree of anthropogenic environmental disturbance under which the highest number of species and records are found. For the latter, the human footprint layer developed by González-Abraham et al. (2015) was utilised, which encompasses five categories of anthropogenic landsurface transformation. These established categories depend on the current impact within the environments, divided into untransformed, low, medium, high and very high. These categories have been determined, based on spatial datasets representing different sources of direct land-surface modification due to human activity: human settlements, cultivated lands (agriculture, forestry plantations and cultivated grasslands), cultivated coasts (mariculture) and roads. The dataset was compiled from vector maps provided by the National Institute of Statistics and Geography of Mexico (INEGI) and supplemented with the digital road map from the Mexican Institute of Transportation (IMT) and ESRI's Mexican road database, both at a scale of 1:200,000. These maps were rasterised with a pixel resolution of 500 m × 500 m, assigning human modification scores to each pixel, based on the intensity and extent of human activity at that location.

Half of Mexico (55.9% of the total land surface) was determined to have a very low human footprint value (category named "untransformed"), suggesting that over half of the country retains its vegetative cover. However, 10.3% was classified with a very high human footprint value. The remaining 33.8% was distributed into intermediate categories: 11.2% with a low footprint, 10.6% with a medium footprint, and 12% with a high footprint (González-Abraham et al. 2015).

A review of specialised literature was carried out, composed of scientific articles that address ant species in Mexico. The main objective was to collect comprehensive information about these species, focusing on their ecological and economic aspects and their impact on human health, regardless of the year of publication of the studies. For this search, keywords that spanned the breadth and depth of these topics were carefully selected. Terms such as "ant species", "ecological importance", "economic significance", "human health impact", "distribution", "records" and "Mexico" were used to ensure thorough collection of relevant information. During this selection process, priority was given to those species that demonstrated a wide geographic distribution or a considerable number of documented records. In relation to health, a verification of the existence of pathogens that were associated with the ant species registered in the country was carried out and their scientific name was validated using the Catalogue of Life (https://www.catalogueoflife.org /).

### Quality control

The scientific name of each ant species was verified using the online catalogue of world ants maintained by Barry Bolton (AntCat) (https://antcat.org/) and the largest online ant database, AntWeb (https://www.antweb.org/). The scientific name of pathogens associated with ant species was validated using the Catalogue of Life (https://www.catalogueoflife.org/). The location of ant species records was corroborated using Google Earth, ensuring that the records fell within the extent considered in the human footprint layer (González-Abraham et al. 2015).

## Geographic coverage

### Description

**Figure 1.**
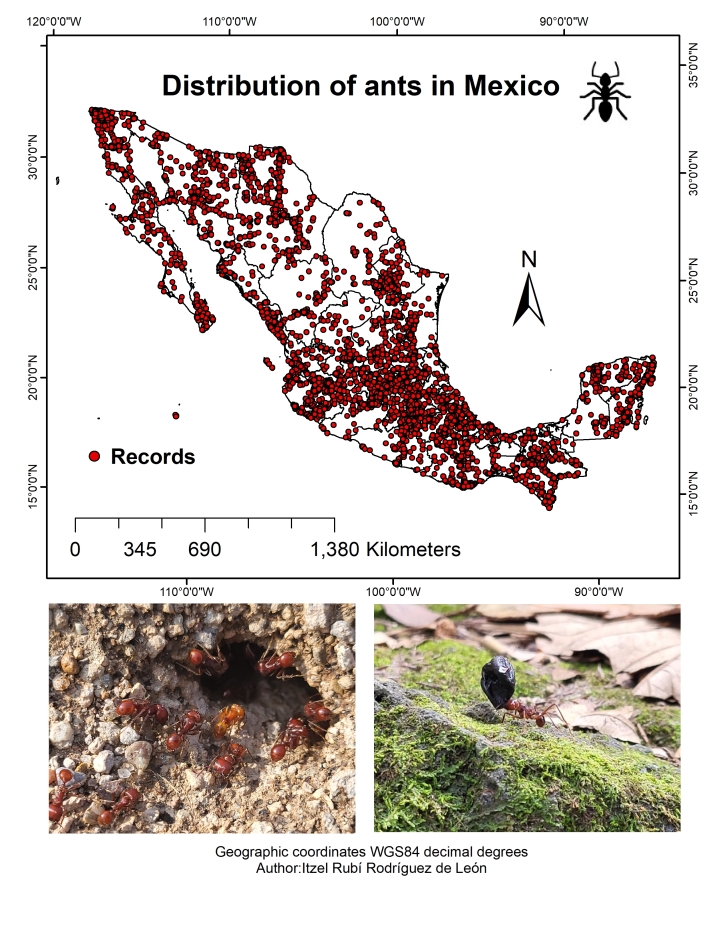
Study area showing the distribution of ant species. The red dots represent the records of ant species in Mexico.

Mexico spans a territorial extent of 1,964,375 km^2^, of which 1,959,248 km^2^ constitute continental land (i.e. mainland within the country) and 5,127 km^2^ comprise island territory (i.e. islands belonging to the country) (Fig. 1). The political division of Mexico consists of 32 states. The human footprint layer incorporates digital vector maps from the National Institute of Statistics and Geography (INEGI) of Mexico and road maps from the Mexican Institute of Transportation (IMT).

## Taxonomic coverage

### Description

**Figure 2.**
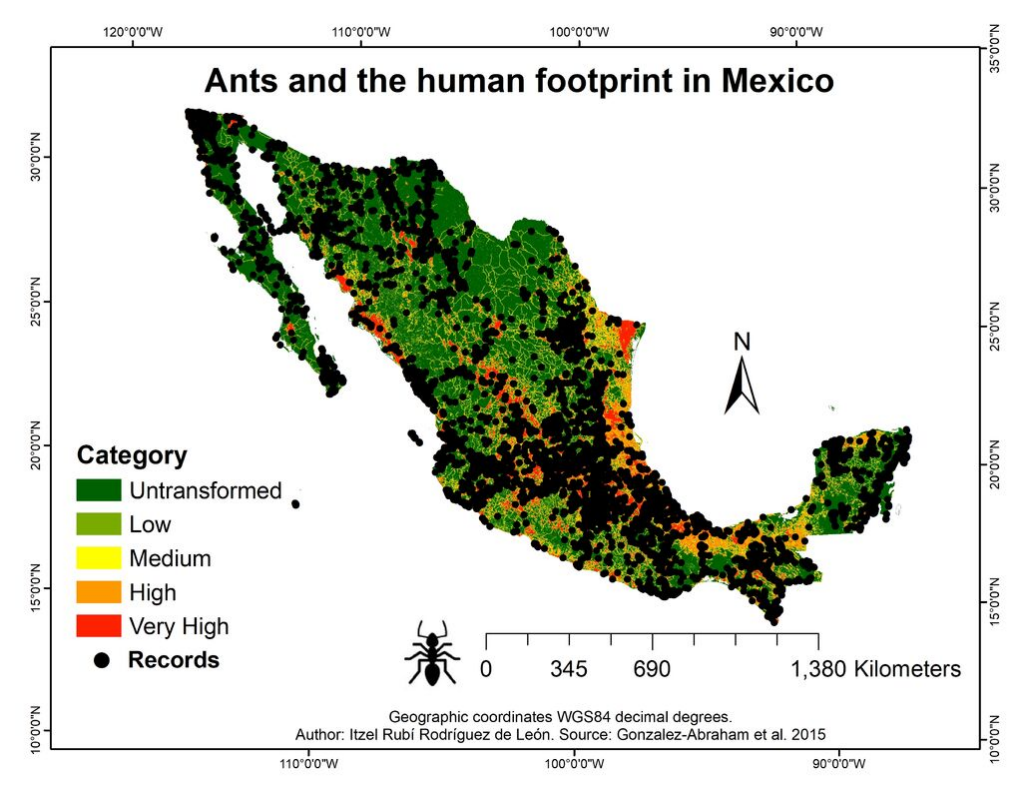
Human footprint in Mexico and the distribution of ants.

The current taxonomy of ants, based on worldwide phylogenetic proposals, has led to significant and important changes in the classification of the family Formicidae. In this study, 33,286 records were obtained for Mexico, corresponding to 1,104 species belonging to 105 genera and 10 subfamilies (Fig. 1, Suppl. material 1). These results represent an increase compared to the numbers in previous studies where 887 species (Dáttilo et al. 2020) and 927 species (Vásquez-Bolaños 2015) were reported. In Mexico, the States with the highest number of ant species are Veracruz (474) and Chiapas (461), while the States with the lowest number of species are Tlaxcala (15) and Aguascalientes (23). The States with the highest number of records are Chiapas (7,509) and Veracruz (6,703). The subfamily with the highest numbers of species (612) and records (18,698) was Myrmicinae, while the subfamily with the lowest number of species was Agroecomyrmecinae, with *Tatuidristatusia*. The States with the highest numbers of records and species in modified environments (high and very high human footprints) are Veracruz (3,546 records and 409 species), Oaxaca (1,211 records and 220 species) and Chiapas (1,035 records and 245 species). The highest numbers of records and species in untransformed environments were found in the States of Chiapas (3,048 records and 289 species), Quintana Roo (932 records and 162 species) and Veracruz (710 records and 144 species) (Table 1 and Fig. 2). Furthermore, it was determined that the most investigated environments amongst ant species studies in the country are those without human impacts, followed by environments with high anthropogenic influence. The species that were mainly found in environments with high human impact are *Attamexicana*, *Pseudomyrmexgracilis* and *Pogonomyrmexbarbatus*, while in untransformed environments, they are *Wasmanniaauropunctata*, *Camponotussericeiventris* and *Strumigenysbrevicornis*. In the case of *W.auropunctata*, despite being an invasive exotic species, it exhibits unique behaviour within untransformed environments. In these areas, where ecosystems remain largely intact and have not been altered by human activity, it has adopted a role more similar to that of a native species. Additionally, in untransformed environments, *W.auropunctata* appears to have a lower population density than in disturbed ecosystems (Salguero et al. 2011). However, it is of vital importance to continue monitoring and controlling the expansion of this invasive species in all ecosystems, with the aim of minimising its negative impact and protecting local biodiversity.

Of the species recorded in the country, 12 are of great relevance in the medical field; six of these cause direct health problems, while eight carry pathogens that cause diseases or discomfort to humans (Tables 2, 3). In ecological terms, ant species play a fundamental role in ecosystems. In the country, there are species that participate in seed dispersal (*Aphaenogasterrudis*) and in the pollination process (species of the genus *Formica*), as well as species that serve as indicators of environmental health, species diversity and population changes (Table 4).

Economically-important species are those considered pests that cause significant losses in crop yields, such as *A.mexicana*, as well as species that have mutualistic interactions with other insect pests. However, it is important to note that there are also species with positive economic impacts, such as *Tapinomamelanocephalum*, an exotic species in the country that is used to control the red spider mite (*Tetranychusurticae*), a pest that affects various crops. Additionally, the presence of *Liometopumapiculatum* has been recorded in Mexico, as well as species belonging to the genus *Atta*, which play a crucial role in various areas as they are consumed as food. These ants provide a valuable source of proteins, vitamins, minerals and essential fatty acids (FAO (Food and Agriculture Organization) 2015, Reyes-Prado et al. 2016, Gallardo-López et al. 2022) (Table 5). From an environmental perspective, the rearing and collection of ants for food purposes is considered a sustainable practice that contributes to the conservation of natural resources and a reduction in environmental impacts.

This study identified one native species (*Solenopsisgeminata)* and four exotic species of significant importance in Mexico. Amongst the exotic species, *T.melanocephalum* stands out as the most prominent and representative species for the country due to its ecological, economic and human health impacts. Furthermore, the importance of the species *Paratrechinalongicornis* and *W.auropunctata* has been determined in both human health and ecosystems. Finally, *Linepithemahumile* stands out for its ecological and economic significance (Tables 2, 3, 4, 5).

## Temporal coverage

### Notes

The distribution of ant species includes records from 1700 to 2022.

## Collection data

### Collection name

National Insect Collection of the Institute of Biology at the National Autonomous University of Mexico (CNIN-UNAM). Collection of the Institute of Applied Ecology at the Autonomous University of Tamaulipas (IEA-UAT) and the National Technological Institute of Mexico (TecNM), Victoria Campus, Tamaulipas.

## Usage licence

### Usage licence

Creative Commons Public Domain Waiver (CC-Zero)

## Data resources

### Data package title

A dataset of the ant distribution in environments with varying human footprints in Mexico.

### Number of data sets

1

### Data set 1.

#### Data set name

A dataset of the ant distribution in environments with varying human footprints in Mexico.

#### Description

The dataset (Suppl. material 1), compiled from the GBIF database and three entomological collections in Mexico from 1970 to 2022, reflects the distribution, species count and records of ant species in environments with varying human footprints. The scientific name of each species was verified using the online world ant catalogue maintained by Barry Bolton (AntCat) (https://antcat.org/) and the largest online ant database, AntWeb (https://www.antweb.org/). The database is presented in Darwin Core format.

**Data set 1.** 

Column label	Column description
ocurrenceID	An identifier for the dwc:Occurrence (as opposed to a particular digital record of the dwc:Occurrence). In the absence of a persistent global unique identifier, construct one from a combination of identifiers in the record that will most closely make the dwc:occurrenceID globally unique.
basisOfRecord	The specific nature of the data record.
associatedReferences	A list (concatenated and separated) of identifiers (publication, bibliographic reference, global unique identifier, URI) of literature associated with the dwc:Occurrence.
institutionCode	The name (or acronym) in use by the institution having custody of the object(s) or information referred to in the record.
collectionCode	The name, acronym, coden or initialism identifying the collection or dataset from which the record was derived.
catalogNumber	An identifier (preferably unique) for the record within the dataset or collection.
kingdom	The full scientific name of the kingdom in which the dwc:Taxon is classified.
phylum	The full scientific name of the phylum or division in which the dwc:Taxon is classified.
class	The full scientific name of the class in which the dwc:Taxon is classified.
order	The full scientific name of the order in which the dwc:Taxon is classified.
family	The full scientific name of the family in which the dwc:Taxon is classified.
subfamily	The full scientific name of the subfamily in which the dwc:Taxon is classified.
scientificName	The full scientific name, with authorship and date information, if known. When forming part of a dwc:Identification, this should be the name in the lowest level taxonomic rank that can be determined. This term should not contain identification qualifications, which should instead be supplied in the dwc:identificationQualifier term.
decimalLatitude	The geographic latitude (in decimal degrees, using the spatial reference system given in dwc:geodeticDatum) of the geographic centre of a dcterms:Location. Positive values are north of the Equator, negative values are south of it. Legal values lie between -90 and 90, inclusive.
decimalLongitude	The geographic longitude (in decimal degrees, using the spatial reference system given in dwc:geodeticDatum) of the geographic centre of a dcterms:Location. Positive values are east of the Greenwich Meridian, negative values are west of it. Legal values lie between -180 and 180, inclusive.
geodeticDatum	The ellipsoid, geodetic datum or spatial reference system (SRS) upon which the geographic coordinates given in dwc:decimalLatitude and dwc:decimalLongitude are based.
coordinateUncertaintyInMetres	The horizontal distance (in metres) from the given dwc:decimalLatitude and dwc:decimalLongitude describing the smallest circle containing the whole of the dcterms:Location. Leave the value empty if the uncertainty is unknown, cannot be estimated or is not applicable (because there are no coordinates). Zero is not a valid value for this term.
individualCount	The number of individuals present at the time of the dwc:Occurrence.
eventDate	The date-time or interval during which a dwc:Event occurred. For occurrences, this is the date-time when the dwc:Event was recorded. Not suitable for a time in a geological context.
day	The integer day of the month on which the dwc:Event occurred.
month	The integer month in which the dwc:Event occurred.
year	The four-digit year in which the dwc:Event occurred, according to the Common Era Calendar.
recordedBy	A person, group or organisation responsible for recording the original dwc:Occurrence.
identifiedBy	A list (concatenated and separated) of the globally-unique identifier for the person, people, groups or organisations responsible for assigning the dwc:Taxon to the subject.
locationRemarks	Comments or notes about the dcterms:Location.
country	The name of the country or major administrative unit in which the dcterms:Location occurs.
stateProvince	The name of the next smaller administrative region than country (state, province, canton, department, region etc.) in which the dcterms:Location occurs.
locality	The specific description of the place.

## Supplementary Material

21C2552C-9151-5DD3-8B94-9AB7E0705309 10.3897/BDJ.11.e109794.suppl1 Supplementary material 1 A dataset of the ant distribution in environments with varying human footprints in Mexico Data type Ocurrences Brief description The dataset, compiled from the GBIF database and three entomological collections in Mexico from 1970 to 2022, reflects the distribution, species count and records of ant species in environments with varying human footprints. The scientific name of each species was verified using the online world ant catalogue maintained by Barry Bolton (AntCat) (https://antcat.org/) and the largest online ant database, AntWeb (https://www.antweb.org/). The database is presented in Darwin Core format. File: oo_880723.zip https://binary.pensoft.net/file/880723 Itzel Rubí Rodríguez de León, Crystian Sadiel Venegas Barrera, Griselda Gaona García, Ausencio Azuara Domínguez, Madai Rosas Mejía

## Figures and Tables

**Table 1.** Numbers of records (rds) and species in each State as impacted by human footprint.

**States**	**Very High**		**High**		**Medium**		**Low**		**Untransformed**	
	**No. rds**	**Nο. species**	**No. rds**	**Nο. species**	**No. rds**	**Nο. species**	**No. rds**	**Nο. species**	**No. rds**	**Nο. species**
Aguascalientes	68	17	17	8	9	5	6	3	5	4
Baja California	101	34	115	47	146	51	74	39	244	78
Baja California Sur	34	20	42	17	51	28	41	20	80	45
Campeche	1	1	30	22	46	38	7	5	234	46
Chiapas	270	124	765	181	1672	277	1754	290	3048	289
Chihuahua	497	76	276	68	433	97	81	35	268	88
Coahuila	62	34	47	35	379	29	14	8	90	28
Colima	31	21	22	14	11	3	3	1	23	18
Ciudad de Mexico	167	32	7	3	8	4	2	2	0	0
Durango	47	18	46	26	11	6	14	7	57	36
Guanajuato	146	20	47	17	22	14	23	11	35	21
Guerrero	197	53	95	43	35	27	25	16	7	7
Hidalgo	270	70	133	53	36	21	25	19	6	5
Jalisco	493	81	145	39	138	47	107	44	180	69
Mexico	192	42	44	19	29	23	17	12	28	12
Michoacan	184	41	87	39	37	17	13	5	33	14
Morelos	442	83	51	28	31	17	25	14	9	7
Nayarit	180	64	56	34	35	26	24	15	88	46
Nuevo Leon	369	74	137	67	108	60	134	54	151	57
Oaxaca	352	130	859	173	1729	202	258	113	132	44
Puebla	293	111	249	120	56	39	35	31	70	43
Queretaro	80	19	60	22	43	22	33	18	21	15
Quintana Roo	210	68	274	88	302	90	285	93	932	162
San Luis Potosi	219	82	176	73	50	28	23	12	47	18
Sinaloa	255	68	359	61	59	23	71	17	37	16
Sonora	145	46	125	37	122	51	59	30	305	84
Tabasco	64	44	25	21	0	0	0	0	2	2
Tamaulipas	429	88	156	72	63	44	203	51	779	76
Tlaxcala	13	11	4	4	1	1	0	0	0	0
Veracruz	1731	281	1815	325	1251	223	1196	214	710	144
Yucatan	87	31	51	25	65	29	31	17	46	21
Zacatecas	32	13	8	7	29	21	4	4	41	20

**Table 2.** Ant species that cause direct harm to human health.

**Species**	**Common name**	**Disease or symptoms**	**Reference**
*Wasmanniaauropunctata*	Fire ant	Keratopathy that can cause leukoma.	(Olivera and Porras-Villamil 2021)
*Solenopsisinvicta*	Red fire ant	Pustules (skin lesions) and allergies.	(Xu et al. 2012)
*Solenopsisgeminata*		Allergic vasculitis (inflammation of blood vessels).	(Do-Nascimento et al. 2020)
*Monomoriumpharaonis*		Allergic reactions.	(Do-Nascimento et al. 2020)
*Trichomyrmexdestructor*		Allergic reactions.	(Olivera and Porras-Villamil 2021)
*Pheidolepallidula*	Barber ant	Alopecia.	(Olivera and Porras-Villamil 2021)

**Table 3.** Species of ants carrying pathogens that affect human health.

**Vector (Species)**	**Pathogen**	**Disease**	**Reference**
*Monomoriumpharaonis*	*Pseudomonasaeruginosa*	Pulmonary or urinary tract infections (kidneys and bladder).	(Garcia and Lise 2013, Do-Nascimento et al. 2020, Newman et al. 2022)
	*Enterococcusfaecalis*	Endocarditis, urinary tract infections and prostitis.	(Garcia and Lise 2013, Fernández et al. 2019, Do-Nascimento et al. 2020, Codelia-Anjum et al. 2023)
	*Enterobactercloacae*	Urinary tract infection or surgical wound infection.	(Garcia and Lise 2013, Do-Nascimento et al. 2020, Jiménez-Guerra et al. 2020)
*Paratrechinalongicornis*	*Sphingomonaspaucimobilis*	Meningitis, arthritis, peritonitis and pneumonia.	(Garcia and Lise 2013, Deveci et al. 2017)
	*Staphylococcussaprophyticus*	Urinary tract infection.	(Orden-Martínez et al. 2008, Garcia and Lise 2013)
	*Stenotrophomonasmaltophilia*	Pneumonia, bronchitis, endocarditis, skin infections and urinary tract infections.	(Garcia and Lise 2013 Campanella et al. 2023)
	*Streptococcusagalactiae*	Bladder infections, bloodstream infections, skin infections, pneumonia and meningitis in babies.	(Garcia and Lise 2013, Coria et al. 2018, Raabe and Shane 2019)
	*Klebsiellapneumoniae*	Nosocomial infections.	(Garcia and Lise 2013)
	*Escherichiacoli*	Diarrhoea.	(Garcia and Lise 2013)
	*Mycobacteriumsmegmatis*	Pulmonary disease, endocarditis, arthritis and skin infections.	(Roxo et al. 2020)
	*Mycobacteriumtuberculosis*	Tuberculosis.	(Roxo et al. 2020)
*Solenopsisgeminata*	*Shigella* spp.	Diarrhoea.	(Simothy et al. 2018)
*Dorymyrmexpyramicus*	*Escherichiacoli*	Diarrhoea.	(Garcia and Lise 2013)
*Linepithemahumile*	*Escherichiacoli*	Diarrhoea.	(Garcia and Lise 2013)
*Wasmanniaauropunctata*	*Pseudomonasaeruginosa*	Pulmonary infections, urinary tract infections (kidneys and bladder) or bone infections.	(Garcia and Lise 2013, Simothy et al. 2018, Newman et al. 2022)
*Tapinomamelanocephalum*	*Enterococcusfaecalis*	Endocarditis (infection of the inner lining of the heart), urinary tract infections and intra-abdominal infections.	(Garcia and Lise 2013, Fernández et al. 2019, Codelia-Anjum et al. 2023)
	*Acinetobacterhaemolyticus*	Nosocomial infections.	(Garcia and Lise 2013, Almasaudi 2018)
*Tetramoriumbicarinatum*	*Pseudomonasaeruginosa*	Pulmonary infections, urinary tract infections (kidneys and bladder) or bone infections.	(Garcia and Lise 2013, Newman et al. 2022)
	*Pseudomonasputida*	Bloodstream infection in neonates, urinary tract infections, pneumonia and sepsis.	(Yoshino et al. 2011, Garcia and Lise 2013)
	*Staphylococcusepidermidis*	Skin infections.	(Garcia and Lise 2013, Chessa et al. 2015)
	*Staphylococcussaprophyticus*	Acute urinary tract infections.	(Orden-Martínez et al. 2008, Garcia and Lise 2013)

**Table 4.** Ecologically-important ant species.

**Species**	**Ecological importance**	**Reference**
*Aphaenogasterrudis*	Seed dispersal.	(Gordon et al. 2019)
*Formicafusca*	Pollinators.	(de-Vega and Gómez 2014)
*Formicaargentea*		
*Cardiocondylaemeryi*		
*Formicaneorufibarbis*		
*Pheidolepallidula*		
*Lasiusalienus*		
*Lasiusniger*		
*Tapinomamelanocephalum*		
*Tapinomasessile*		
*Nylanderiavividula*		
*Prenolepisimparis*		
*Ecitonburchellii*	Indicators of bird diversity/Indicators of ecological health.	(Pérez-Espona 2021)
*Labiduspraedator*		
*Wasmanniaauropunctata*	Negative indicator of dry forest ant richness.	(Wetterer and Porter 2003, Salguero et al. 2011, Murguía-González et al. 2018)
*Pogonomyrmeximberbiculus*	Indicators of semi-arid environments where there is little vegetation cover, such as shrublands and grasslands.	(Rodríguez-de-León et al. 2019)
*Pogonomyrmexbarbatus*		
*Tetramoriumspinosum*		
*Solenopsisgeminata*	Native species with invasive behaviour that negatively impacts vertebrate species, such as the loggerhead sea turtle (*Carettacaretta*), as well as bird nests and iguanas. Additionally, it harms invertebrates by displacing other ant species and preying on butterfly eggs or snails.	(Risch and Carroll 1982, Nafus and Schreiner 1988, Moulis 1996, Yusa 2001)
*Linepithemahumile*	Exotic and invasive species that can cause disruptions in native invertebrate fauna to the extent of altering the assemblage of native species.	(Kroll et al. 1973, Armbrecht and Ulloa-Chacón 2003, Allen et al. 2004, Bution et al. 2010)
*Paratrechinalongicornis*		
*Solenopsisinvicta*		
*Tapinomamelanocephalum*		

**Table 5.** Economically-important ant species.

**Species**	**Economic importance**	**Reference**
*Dorymyrmexflavus*	Mutualistic interactions with the mealybugs that are pests of sugar-cane.	(Murguía-González et al. 2018)
*Tapinomamelanocephalum*	Biological control of the red spider mite (*Tetranychusurticae*), a pest of various crops. Interactions with populations of phloem-feeding hemipteran insects, such as aphids, scale insects and mealybugs.	(Murguía-González et al. 2018)
*Linepithemahumile*	Causes severe indirect damage to crops as it feeds on the honeydew secreted by various aphids.	(Murguía-González et al. 2018)
*Liometopumapiculatum*	The larvae are used for human consumption.	(Figueroa-Sandoval et al. 2018)
*Attamexicana*	Pest that causes significant losses in the yield of forest crops, citrus fruits, fruits, cocoa, coffee, corn and pastures. The queens of this species (known as chicatanas) are used for human consumption.	(Reyes-Prado et al. 2016)
*Solenopsisgeminata*	Crop pest. Interaction with populations of hemipterans, such as pseudococcids.	(Blanco 2004, Wilson 2005)
